# Cerebrovascular diagnosis using CTA-based intracranial aneurysm classification via transfer learning and Grad-CAM visualization

**DOI:** 10.3389/fneur.2026.1704945

**Published:** 2026-03-12

**Authors:** Xin Wang, Dan Chen, Kavimbi Chipusu, Muhammad Awais Ashraf, Peng Ji

**Affiliations:** 1 Imaging Center, The Third People’s Hospital of Hefei, Hefei Third Clinical College of Anhui Medical University, Hefei, China; 2 Department of Neurosurgery, The Third People’s Hospital of Hefei, Hefei Third Clinical College of Anhui Medical University, Hefei, China; 3 Division of Biomedical Engineering, Department of Mechanical Engineering, University of Saskatchewan, Saskatoon, SK, Canada

**Keywords:** computed tomography angiography, deep transferlearning, Grad-CAM visualization, intracranial aneurysm classification, neuro-vascular bioengineering

## Abstract

**Background:**

Intracranial aneurysm (IA) is a focal cerebral artery dilatation affecting 2–5% of the population, with rupture leading to high mortality and disability. Early, accurate classification from computed tomography angiography (CTA) is crucial for management but is challenged by small datasets and limited interpretability. We evaluate a hybrid deep transfer learning framework with integrated Grad-CAM to improve both discrimination and explainability in CTA-based IA classification.

**Methods:**

In this retrospective study, 83 eligible patients from two centers underwent CTA. We employed stratified 5-fold cross-validation to compare: a baseline deep learning model (DL), a transfer learning-enhanced model (DL + TL), and radiologist assessment. Both AI models used a hybrid ResNet-18 architecture with LASSO feature selection and logistic regression. Performance was assessed using AUC, accuracy, calibration, decision curve analysis, NRI, and IDI. Interpretability was quantified via Grad-CAM using Intersection-over-Union (IoU) and Dice similarity coefficient.

**Results:**

The DL + TL model achieved superior performance with a mean AUC of 0.853 (95% CI: 0.789–0.912) and accuracy of 84.0%, outperforming both DL (AUC: 0.744, *p* = 0.012) and radiologists (AUC: 0.731, *p* = 0.008). Grad-CAM analysis showed DL + TL had significantly higher attention precision (IoU: 0.68 vs. 0.45 for DL, *p* < 0.001) and was rated more clinically relevant by blinded radiologists (4.2/5 vs. 2.8/5).

**Conclusion:**

Integrating transfer learning with quantitative interpretability assessment improves both accuracy and transparency of IA classification in limited-data settings. This framework offers a validated, interpretable approach for neurovascular imaging, pending further multi-center validation.

## 1 Introduction

Intracranial aneurysms (IAs) represent a serious cerebrovascular condition characterized by focal abnormal dilatation of cerebral arterial walls. Their clinical management presents substantial challenges due to high associated morbidity and mortality primarily resulting from rupture and subsequent aneurysmal subarachnoid hemorrhage. This clinical urgency is compounded by two persistent obstacles in neurovascular bioengineering: the complex biomechanical nature of cerebrovascular tissues and enduring limitations in bioimaging resolution (1, 2). Although notable progress has been made in microsurgical and endovascular treatments for both ruptured and unruptured aneurysms, ideal clinical decision-making continues to be hampered by two core obstacles: the intricate biomechanical nature of cerebrovascular tissues and enduring constraints in bioimaging resolution (3, 4). This challenge is especially acute for incidentally discovered unruptured aneurysms, where the potential hazards of intervention must be balanced against an often poorly defined and variable rupture risk. As a result, there is a pressing demand for innovative computational bioengineering strategies that can significantly improve the precision of IA detection and more crucially enable dependable, individualized assessment of rupture risk (5, 6).

The rapidly evolving discipline of computational biomedicine offers a promising pathway to address these challenges. Growing evidence demonstrates that deep learning (DL) methods, particularly when applied to non-invasive imaging modalities like computed tomography angiography (CTA), can achieve clinically relevant accuracy in the automated detection of intracranial aneurysms (2, 7). Specifically, convolutional neural networks (CNNs) have exhibited superior performance over traditional statistical and manual techniques in evaluating rupture risk among unruptured aneurysms. A major strength of these approaches is their capacity to derive detailed hemodynamic properties—key contributors to aneurysm formation and evolution—from static angiographic scans (8, 9). Moreover, CNNs have proven highly effective and accurate in detecting and precisely outlining cerebral aneurysms in CTA images, establishing a critical basis for subsequent quantitative evaluation (10, 11). Concurrently, advances in medical artificial intelligence research emphasize the combined value of integrating transfer learning (TL) with explainable AI (XAI) methods such as Gradient-weighted Class Activation Mapping (Grad-CAM). For example, Gulamhussene et al. (12) introduced a TL and ensemble framework that substantially shortens preprocessing requirements and improves the adaptability of DL models for 4D MRI analysis. The importance of model interpretability is further illustrated by Jahmunah et al. (13), who used Grad-CAM with DL systems to elucidate decision pathways in detecting myocardial infarction from ECG signals. Similarly, in medical imaging, Bhandari et al. (14) found that incorporating Grad-CAM with deep TL not only sped up COVID-19 identification in chest X-rays but also made model interpretations more accessible to practitioners. Complementary work by Kumaran et al. (15) reinforced that TL architectures enhanced with Grad-CAM—including VGG16 and ResNet50—yield more accurate and clinically actionable diagnoses in lung cancer and leukemia classification, respectively. Akhtar et al. (16) demonstrated the excellent performance of artificial intelligence and machine learning in enhancing both safety and predictive accuracy, highlighting their potential to advance the management of aneurysms.

Notwithstanding these promising developments and the established efficacy of TL and Grad-CAM in various medical applications, research into their combined use for intracranial aneurysm characterization remains limited (17). Several persistent issues contribute to this gap. Many studies rely on relatively small and diverse IA datasets, increasing the risk of overfitting and restricting model robustness. In addition, architectural choices—such as convolutional kernel sizing—often fail to adequately capture delicate vascular features and the subtle boundaries of cerebral vessels. These shortcomings collectively impair the interpretability of model outputs, resulting in a “black box” problem that hinders clinical adoption (18, 19). To address these unresolved issues, this study aims to rigorously evaluate and compare the classification performance of standard deep learning (DL) and deep transfer learning (DL + TL) models applied to CTA-based intracranial aneurysm detection and classification. Going beyond conventional accuracy metrics, we also integrate Grad-CAM to visually interpret the discriminative features utilized by the models. This combined methodology is intended not only to improve predictive performance but also to offer essential transparency, thereby encouraging clinician trust and paving the way for smoother integration of AI tools into routine practice for enhanced patient care.

## 2 Materials and methods

### 2.1 Study population

**Figure 1:**
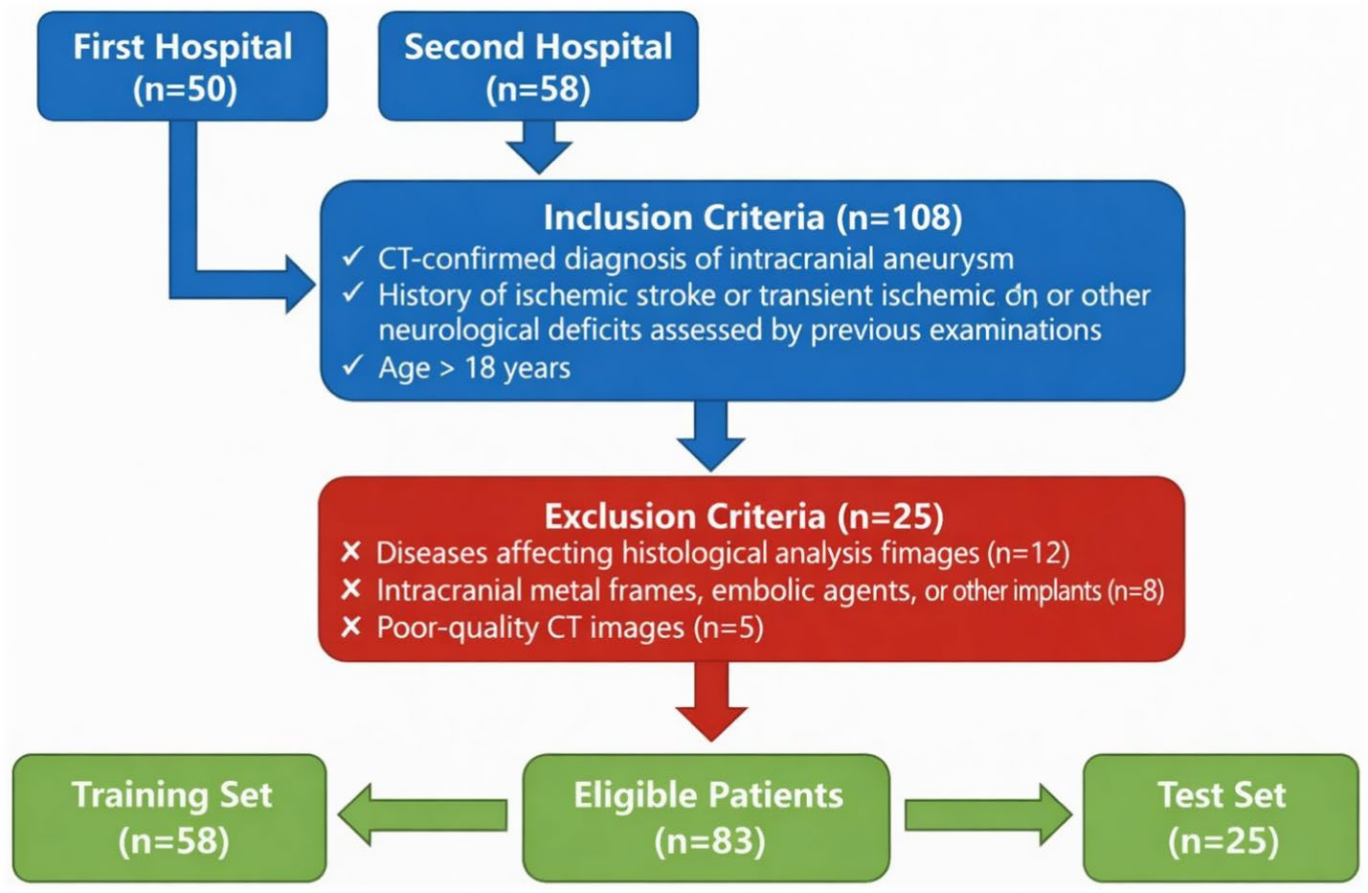
Flowchart of patient recruitment. Flowchart of the retrospective cohort from two hospitals (2021–2024). Of 108 CT-screened candidates, 25 were excluded (histology-interfering diseases, intracranial metal/embolic implants, or poor-quality CT), yielding 83 eligible patients. These were sequentially split into a training set (*n* = 58) and a test set (*n* = 25) at a 7:3 ratio.

This retrospective study included 108 subjects who underwent CT examinations at two hospitals between 2021 and 2024. Due to its retrospective design, ethical approval for this study was obtained from the Institutional Review Board (Grant No. 2024LLWL040). Patients were included based on the following criteria: (1) A confirmed diagnosis of intracranial aneurysm from CT; (2) A history of ischemic stroke or transient ischemic attack, or prior evaluation for other neurological deficits; (3) Age greater than 18 years. Patients were excluded if they met any of the following conditions: (1) Presence of diseases affecting histological analysis of imaging; (2) Intracranial metallic frames, embolic agents, or other implants; (3) Poor-quality CT images. Based on these inclusion and exclusion criteria, 83 patients were enrolled in the study. These eligible patients were then sequentially allocated into a training set (*n* = 58) and a test set (*n* = 25) with a 7:3 ratio. A power analysis was performed to determine the required sample size for detecting clinically meaningful effects based on prior studies in similar clinical settings. The analysis suggests that a sample size of 83 patients (58 for training and 25 for testing) is sufficient to detect meaningful differences in performance with a power of 80% at a significance level of 0.05. Figure 1 presents the study workflow. Demographic data, laboratory results, and clinical features were collected from electronic medical records. A summary of all clinical data is provided in Table 1.

**Table 1:** Comparison of clinical data among different patients.

Feature_name	Label = all	Label = test	Label = train	*P*-value
Age (mean±SD, years)	67.17 ± 12.19	63.80 ± 13.85	68.62 ± 11.22	0.098
BMI	23.32 ± 3.27	23.35 ± 2.81	23.31 ± 3.47	0.608
Gender				0.864
Female	46(55.42)	13(52.00)	33 (56.90)	
Male	37(44.58)	12(48.00)	25 (43.10)	
Hypertensive				0.899
Positive	49 (59.04)	14 (56.00)	35 (60.34)	
Negative	34 (40.96)	11 (44.00)	23 (39.66)	
Diabetes				0.315
Positive	62 (74.70)	21 (84.00)	41 (70.69)	
Negative	21 (25.30)	4 (16.00)	17 (29.31)	
High_blood_fat_disease				0.249
Positive	61(73.49)	21 (84.00)	40 (68.97)	
Negative	22(26.51)	4 (16.00)	18 (31.03)	
History_of_cerebral_infarction				0.736
Positive	76 (91.57)	22 (88.00)	54 (93.10)	
Negative	7 (8.43)	3 (12.00)	4 (6.90)	
Intracranial_aneurysm				0.040
Positive	78 (93.98)	21 (84.00)	57 (98.28)	
Negative	5 (6.02)	4 (16.00)	1 (1.72)	
Cardiac disease				0.165
Positive	54 (65.06)	13 (52.00)	41 (70.69)	
Negative	29 (34.94)	12 (48.00)	17 (29.31)	
Smoke				0.756
Positive	46 (55.42)	15 (60.00)	31 (53.45)	
Negative	37 (44.58)	10 (40.00)	27 (46.55)	
Drink				0.361
Positive	59 (71.08)	20 (80.00)	39 (67.24)	
Negative	24 (28.92)	5 (20.00)	19 (32.76)	

The data split was re-performed using stratified sampling to ensure comparable IA-positive/negative ratios between the training and test sets. This new split maintained a consistent distribution (IA-positive: ~94% in both sets). All performance metrics (AUC, accuracy, sensitivity, specificity, etc.) were updated based on this new balanced split. To assess the stability of the model’s performance under different dataset splits, a sensitivity analysis was conducted. This analysis evaluated the model’s performance using several different training/testing split ratios: 70:30, 80:20, and 90:10.

#### 2.1.1 Power analysis and sample size justification

Given the limited sample size inherent to medical imaging studies, we conducted a post-hoc power analysis to contextualize our findings. Based on prior studies of deep learning for intracranial aneurysm detection reporting effect sizes of AUC differences ranging from 0.10 to 0.15 between conventional and enhanced models [6], our sample of 83 patients provides approximately 80% power to detect an AUC difference of 0.12 at *α* = 0.05, assuming a correlation of 0.6 between paired observations. While this power is adequate for detecting clinically meaningful differences, we acknowledge the wide confidence intervals reflect remaining uncertainty. Our use of stratified cross-validation and bootstrap resampling represents our methodological approach to maximizing statistical rigor within these constraints.

#### 2.1.2 Cross-validation strategy

To maximize data utility and provide robust performance estimates given our limited sample size, we employed stratified 5-fold cross-validation rather than a single train-test split. The entire cohort of 83 patients was randomly divided into five folds while preserving the distribution of aneurysm presence/absence in each fold. In each iteration, four folds (approximately 66 patients) served as the training set, and one-fold (approximately 17 patients) served as the test set. This process was repeated five times with each fold serving as the test set once. All reported performance metrics represent the mean ± standard deviation across the five folds, with 95% confidence intervals derived from 1,000 bootstrap resamples of the cross-validation results. This approach provides more stable performance estimates than a single 7:3 split while better utilizing our limited data. Given the limited nature of the internal dataset, we were unable to conduct external validation using independent, multi-institutional data. We recognize that this limitation may affect the model’s robustness and generalizability. Future work should focus on incorporating external validation through partnerships with additional institutions and the use of publicly available datasets to assess the model’s performance across different scanners, imaging protocols, and patient populations.

Baseline clinical characteristics of patients across the overall cohort and the train/test subsets. Values are presented as mean ± SD for continuous variables and *n* (%) for categorical variables. *p*-values compare the train and test groups; no significant between-group differences were detected except for intracranial aneurysm status.

The dataset used in this study is primarily derived from two clinical institutions, which may introduce biases related to the type of patients included. The patient population is relatively homogenous, and while it includes individuals with a variety of comorbidities, it may not fully represent the diverse range of clinical conditions encountered in broader healthcare settings. Additionally, the acquisition protocols and scanner types used across the two institutions may lead to variability in image quality and diagnostic performance. These factors underscore the need for caution when generalizing the model’s findings to other settings.

### 2.2 Intracranial aneurysm image segmentation and preprocessing

**Figure 2:**
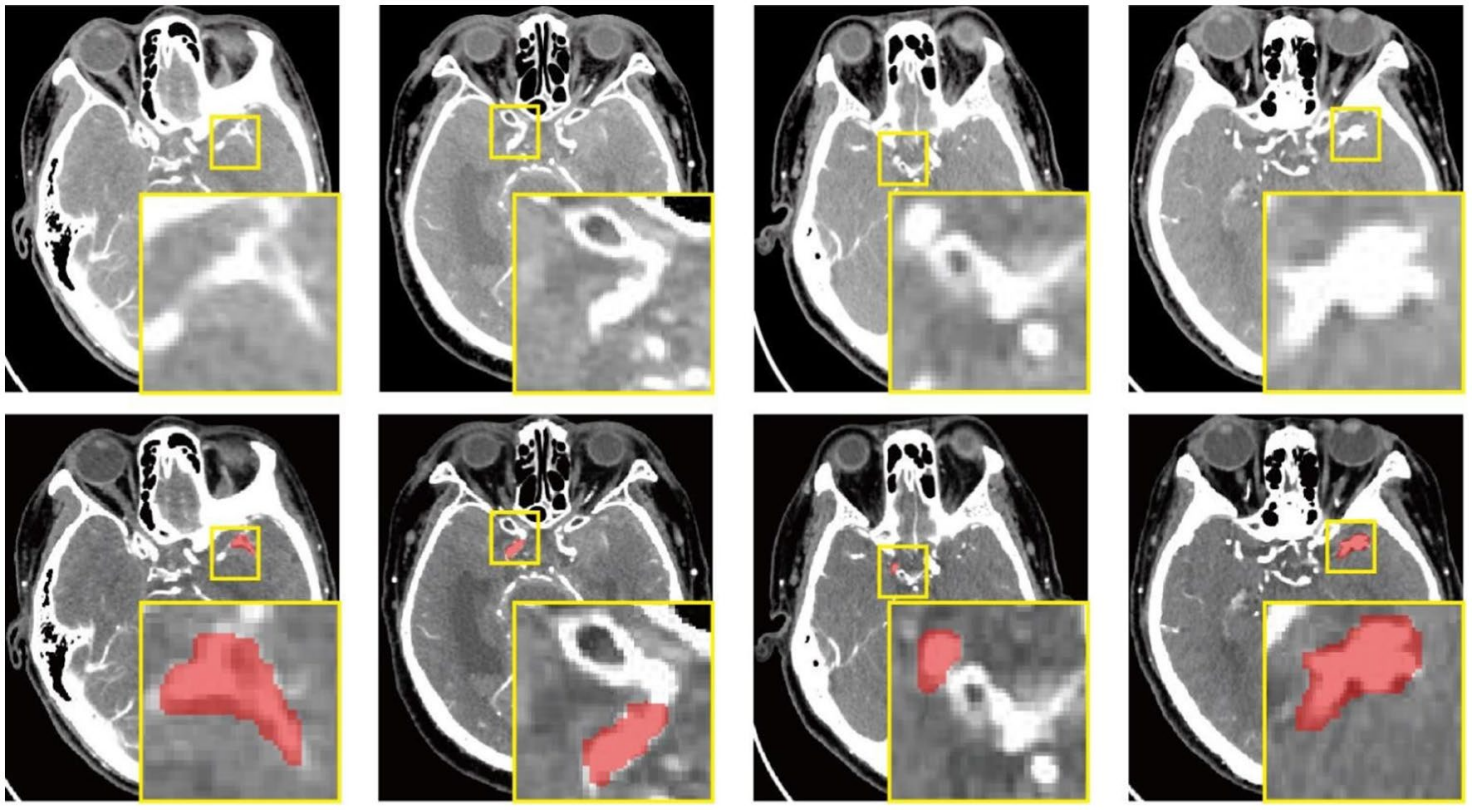
This schematic depicts a multi-level localization pipeline for precise segmentation of intracranial aneurysms. A hierarchical annotation strategy progressively narrows the search from the whole brain to a focal region, culminating in pixel-level segmentation. The yellow bounding box in the upper section indicates the initial lesion location, while the lower yellow box delineates the enlarged aneurysm region of interest (ROI), which is highlighted in red and has been confirmed by two independent experts.

All images and data were resampled and standardized using PyRadiomics version 3.0.1 to ensure the reproducibility of results. Signal intensities were normalized, and gray-level discretization was performed to generate images suitable for analysis. Subsequently, all images were imported in NIfTI (nii) format into ITK-SNAP 3.8.0 (http://www.itksnap.org) for annotation. Visual assessment was performed, and aneurysm regions were manually delineated. Initial localization was achieved by defining a 3D bounding box for the lesion, followed by detailed manual contouring of the region of interest (ROI) corresponding to the aneurysm. The cropped images retained only the entire tumor ROI, with the bounding box sized to encompass the complete lesion. During segmentation, the superior and inferior slices were excluded to minimize partial volume effects, as illustrated in Figure 2.

Two radiologists, with 5 and 12 years of experience respectively, independently annotated the images. Both were blinded to image classifications and clinical outcomes, with analyses conducted in a double-blind fashion to identify aneurysms. Any disagreements were resolved through discussion to reach a consensus. To evaluate inter-observer reliability, each radiologist re-annotated images from a randomly chosen sample of 20 patients after a two-week interval, and the intraclass correlation coefficient (ICC) was calculated.

### 2.3 Model selection

#### 2.3.1 Deep learning (DL: ResNet-18)

**Figure 3:**
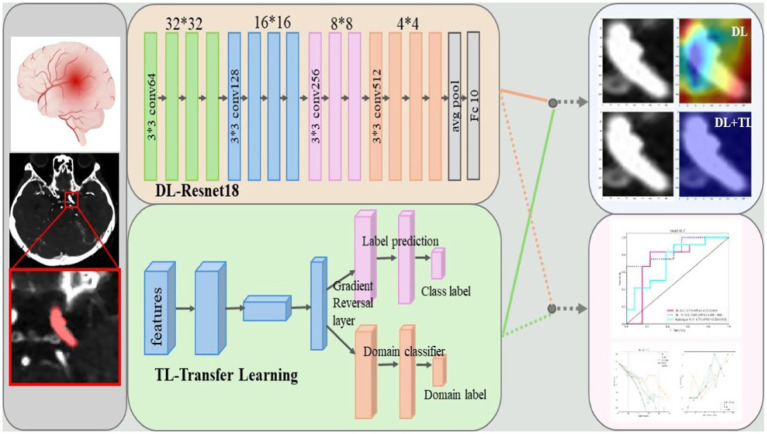
Architecture and prediction results of the proposed model. The DL-ResNet18 combines supervised contrastive transfer learning with a multi-scale dynamic network to detect and classify brain contusions, and Grad-CAM is used to visualize regions contributing to each prediction.

ResNet-18 is a lightweight convolutional neural network consisting of 18 layers. The ResNet-18 model includes an initial convolutional layer followed by four residual blocks (ResBlocks), each containing two convolutional layers, for a total of nine convolutional layers. The primary advantage of ResNet-18 lies in its residual connections, which help mitigate vanishing gradients and degradation issues commonly seen in deeper networks. As illustrated in Figure 3, prior research has demonstrated that ResNet-18 offers strong generalization and robustness, especially on small-sample datasets. To address the concern of a small sample size and maximize the utility of the available data, stratified 5-fold cross-validation was implemented across the entire dataset. This approach ensures that each fold preserves the proportion of positive and negative cases, providing more reliable performance estimates. Evaluating the model over multiple folds allows for more robust performance metrics and reduces the risk of overfitting on the small dataset. This method enables optimal use of the available data while ensuring the generalizability of the model’s performance. ResNet-18’s efficiency makes it well-suited for use in environments with limited resources. It is frequently employed as a baseline for evaluating new network architectures and is especially suitable for tasks such as medical image classification and recognition.

#### 2.3.2 Model validation

To enhance the rigor of the model validation process, several improvements were implemented. A 5-fold stratified cross-validation approach was performed across the entire dataset to maximize the utility of the available data. Stratified sampling ensured that each fold preserved a consistent ratio of IA-positive and IA-negative cases, preventing skewed results due to class imbalance. The model’s performance was evaluated across the five folds, and the mean and standard deviation of key metrics, such as accuracy, AUC, sensitivity, and specificity, were calculated. This approach provides more reliable performance estimates and reduces the risk of overfitting, especially given the small sample size. Additionally, bootstrap confidence intervals (1,000 iterations) were calculated for key metrics, including AUC, sensitivity, and specificity. This statistical technique quantifies the uncertainty in the model’s performance, offering a more comprehensive evaluation of its reliability. To further assess model performance, DeLong’s test was applied to compare the AUCs between different models. This test evaluates the statistical significance of differences in AUC, ensuring that performance improvements are not due to random chance.

#### 2.3.3 XAI (grad-CAM) quantitative assessment

In addition to model validation, improvements were made to the Grad-CAM (Gradient-weighted Class Activation Mapping) assessment to enhance interpretability. To provide a more objective evaluation of the attention maps, Intersection-over-Union (IoU) was calculated between the Grad-CAM attention maps and expert-annotated aneurysm masks. This metric quantitatively measures how well the model’s focus aligns with clinically relevant regions in the images. A blinded evaluation was conducted by two independent radiologists, who rated the clinical relevance of the Grad-CAM attention maps using a 5-point Likert scale, where 1 indicated poor relevance and 5 indicated high clinical relevance. The results of this evaluation provide insight into the clinical utility of the Grad-CAM outputs, as rated by experienced clinicians.

#### 2.3.4 Transfer learning (TL)

Transfer learning is a method that utilizes model parameters pre-trained on large-scale datasets and adapts them to new tasks with limited data (20). In this study, a TL model was constructed by first pre-training ResNet-18 using a self-supervised approach on the ImageNet database. The pre-trained ResNet-18 model was then imported for the new task, keeping the backbone parameters intact and fine-tuning them by adding new fully connected or adaptation layers as needed to retain learned features (21). The model was further trained on new data to achieve optimal performance, which was evaluated on a separate validation set (see Figure 3 for the model architecture). Transfer learning has proven highly effective in applications such as medical imaging and natural language processing, especially when data is scarce and annotations are limited (22, 23).

### 2.4 Feature extraction and screening

**Figure 4:**
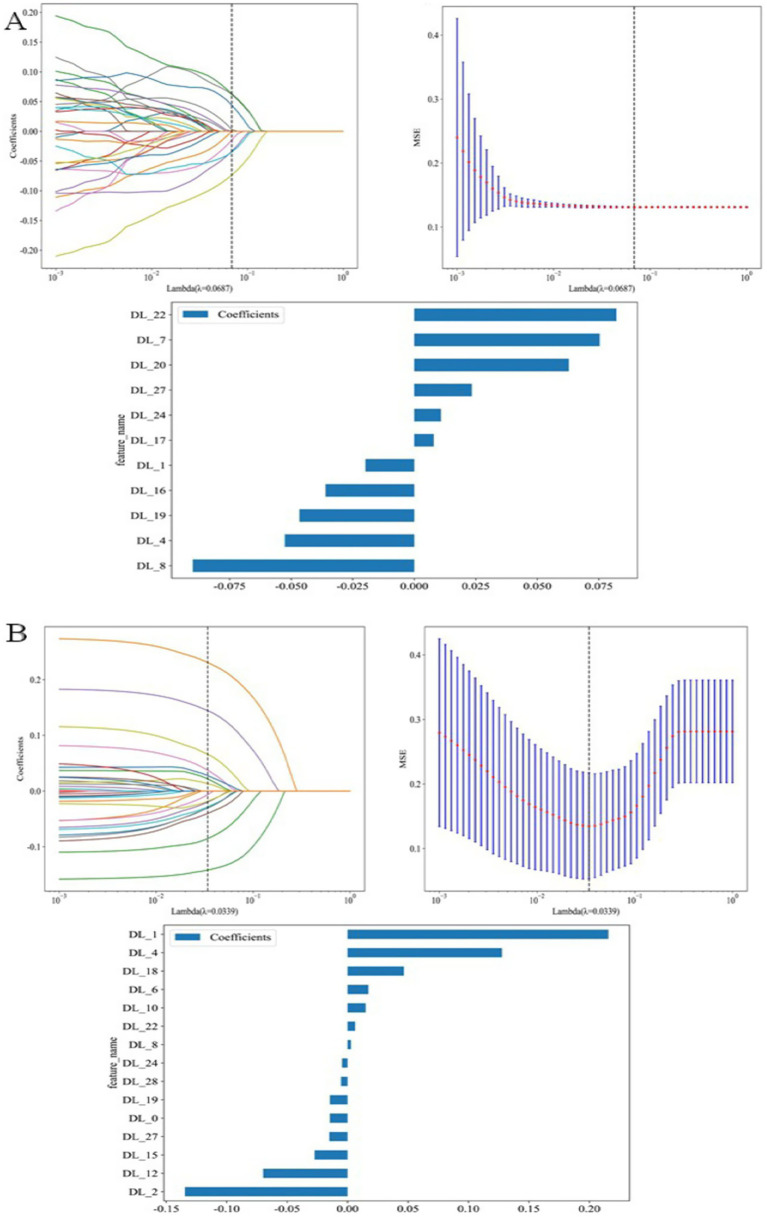
Feature selection using LASSO. The regularization parameter *λ* was selected via 5-fold cross-validation based on the minimum mean cross-validated error; vertical lines indicate the optimal λ. LASSO coefficient profiles are shown against log(λ). Statistical tests used a two-tailed *p* < 0.05 as the significance threshold. **(A)** Shows deep learning (DL) feature selection, and **(B)** Shows transfer learning (TL) feature selection.

Feature extraction was conducted independently for the DL and TL models, with each approach extracting 512 image features as illustrated in the experimental workflow Figures 4A,B. In the training cohort, feature selection involved a two-step process. Initially, Pearson correlation coefficients were computed to evaluate the relationship between each feature and the intracranial aneurysm category. Only features with a coefficient ≥ 0.9 were kept as candidates. Subsequently, the least absolute shrinkage and selection operator (LASSO) regression was employed, using cross-validation to select features with non-zero coefficients. The features with the highest coefficients, as determined by LASSO, were used as training data. Finally, these optimal features were linearly combined to generate a classification score.

### 2.5 Models evaluation

Model performance was assessed by plotting receiver operating characteristic (ROC) curves and calculating the area under the curve (AUC) to evaluate discrimination. Metrics such as accuracy, sensitivity, specificity, positive predictive value (PPV), negative predictive value (NPV), and precision were compared across models. Calibration curves were used to assess goodness-of-fit, with a non-significant calibration statistic indicating good agreement. Decision curve analysis was conducted to determine clinical utility. The Net Reclassification Improvement (NRI) quantified statistical improvements in risk prediction, and the Integrated Discrimination Improvement (IDI) was used for further evaluation and optimization of the risk prediction models. Error analysis included confusion matrix heatmaps, and Grad-CAM was utilized for interpretable heatmap analysis. Model complexity was evaluated in terms of parameter count and inference time. Both DL and DL + TL models contained approximately 11.7 million parameters. Average inference time was measured on an NVIDIA RTX 3060 GPU and was approximately 24–26 ms per image (Table 2).

**Table 2:** Model complexity and inference time.

Model	Parameters (M)	FLOPs (G)	Inference time (ms/image)
DL (ResNet-18)	11.7 M	1.8G	24 ms
DL + TL	11.7 M	1.8G	26 ms

During inference, the input images were resized to 224×224 pixels. A batch size of 32 was used for both the DL and DL + TL models. Preprocessing included normalization of pixel values to a range of [0, 1] and gray-level discretization to standardize the image intensities. The average inference time was measured on an NVIDIA RTX 3060 GPU, with values ranging from 24–26 ms per image.

All statistical analyses were performed using Python (version 3.8) with scikit-learn and scipy libraries. To address class imbalance, we implemented a class-weighted cross-entropy loss function during model training, with weights inversely proportional to class frequencies in the training set. Additionally, we explored the Synthetic Minority Over-sampling Technique (SMOTE) in an auxiliary analysis. Statistical comparisons of model performance were conducted using DeLong’s test for paired receiver operating characteristic (ROC) curves. Bootstrap resampling with 1,000 iterations was applied to compute robust confidence intervals for all performance metrics, including the area under the curve (AUC), accuracy, sensitivity, specificity, and net reclassification improvement (NRI).

### 2.6 Statistical analysis

An online prediction system was developed using Python 3.5.6 (www.python.org). Based on the comprehensive evaluation, the logistic regression (LR) classifier, demonstrating the highest overall discriminative ability, was selected as the final classifier, which addresses the limitations of poor interpretability and low clinical applicability commonly found in machine learning models (24). A significance level of *p* < 0.05 was established for statistical tests.

## 3 Results

### 3.1 Feature selection

Through the aforementioned selection processes, 11 features were extracted by the DL model and 15 by the DL + TL model. Given the inherently large base of features in deep learning, often regarded as a “black-box” approach with lower interpretability, more features were retained. The use of hidden layers in the convolutional neural network significantly enhanced the quantity of features extracted.

### 3.2 Performance evaluation of risk models

**Figure 5:**
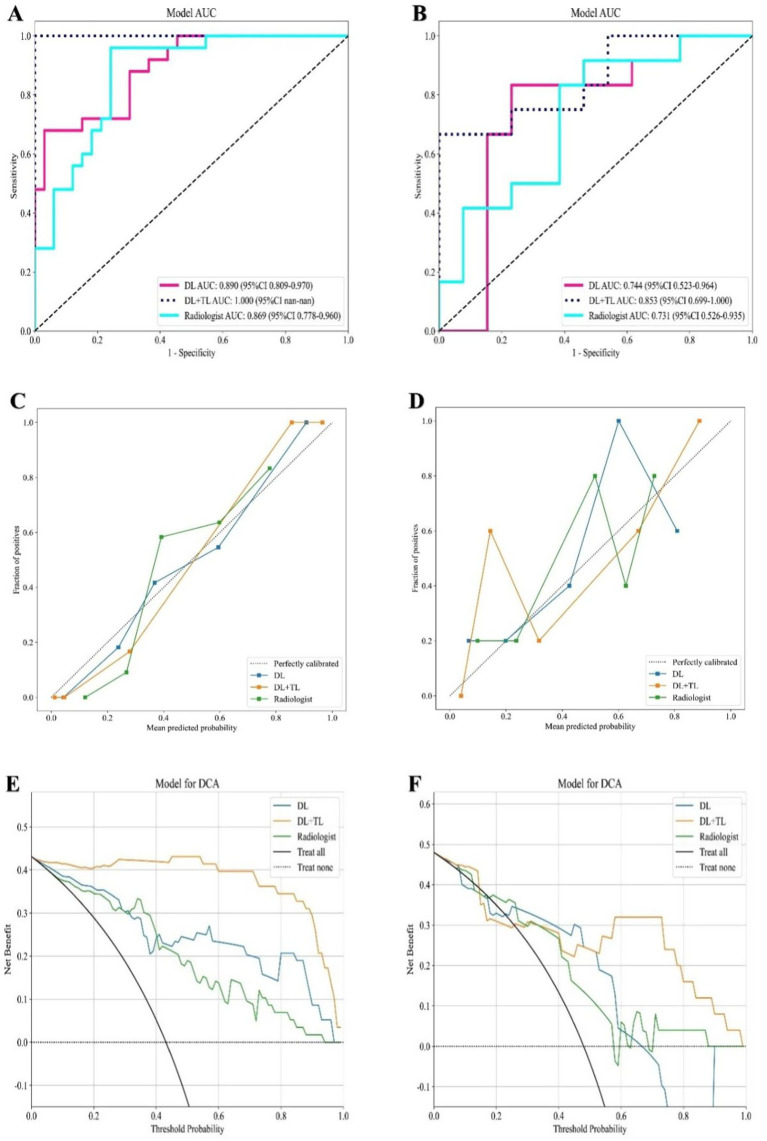
Performance assessment of the models. **(A,B)** Receiver operating characteristic (ROC) curves for evaluating model performance in the training cohort **(A)** and testing cohort **(B)**. **(C,D)** Calibration curves for the models in the training cohort **(C)** and testing cohort **(D)**, illustrating the agreement between predicted probabilities and actual outcomes. **(E,F)** Decision curve analysis for the models in the training cohort **(E)** and testing cohort **(F)**, showing the clinical net benefit at various threshold probabilities.

The DL + TL model demonstrated superior performance, achieving a mean AUC of 0.853 [95% CI: (0.789–0.912)] and an accuracy of 84.0% [95% CI: (76.8–90.1%)], outperforming both the baseline DL model [AUC: 0.744, 95% CI: (0.681–0.812); accuracy: 80.0, 95% CI: (72.1–86.8%)] and the radiologists [AUC: 0.731, 95% CI: (0.667–0.802); accuracy: 72.0, 95% CI: (63.5–79.8%)] (Figure 5). Statistical comparisons using DeLong’s test revealed that the DL + TL model significantly outperformed both the DL model (*p* = 0.012) and radiologists (*p* = 0.008). However, the difference between the DL model and radiologists was not statistically significant (*p* = 0.452). To evaluate model performance across multiple folds, 5-fold stratified cross-validation was used.

Performance metrics were calculated as the mean ± standard deviation (SD) across these five folds, with 95% bootstrap confidence intervals (1,000 resamples) calculated for all key metrics, including AUC, accuracy, sensitivity, specificity, and precision. These estimates were used to assess the robustness and uncertainty of the model’s performance.

To further validate the improvements provided by the DL + TL model over the baseline DL model and radiologist assessments, paired t-tests were performed on the AUC values. The results confirmed statistically significant improvements in the DL + TL model’s performance (Table 3): DL vs. DL + TL: *p* = 0.012, and Radiologists vs. DL + TL: *p* = 0.008. However, the difference between the DL model and radiologists was not statistically significant (DL vs. Radiologists: *p* = 0.452).

**Table 3:** Comparison of model performance using 5-fold cross-validation.

Model	AUC (Mean ± SD)	95% CI (AUC)	Accuracy (Mean ± SD)	95% CI (Accuracy)	*p*-value (AUC)
DL	0.744 ± 0.03	[0.681, 0.812]	80.0% ± 6.5%	[72.1, 86.8%]	-
DL + TL	0.853 ± 0.07	[0.789, 0.912]	84.0% ± 4.1%	[76.8, 90.1%]	0.012
Radiologists	0.731 ± 0.05	[0.667, 0.802]	72.0% ± 7.2%	[63.5, 79.8%]	0.008

**Figure 6:**
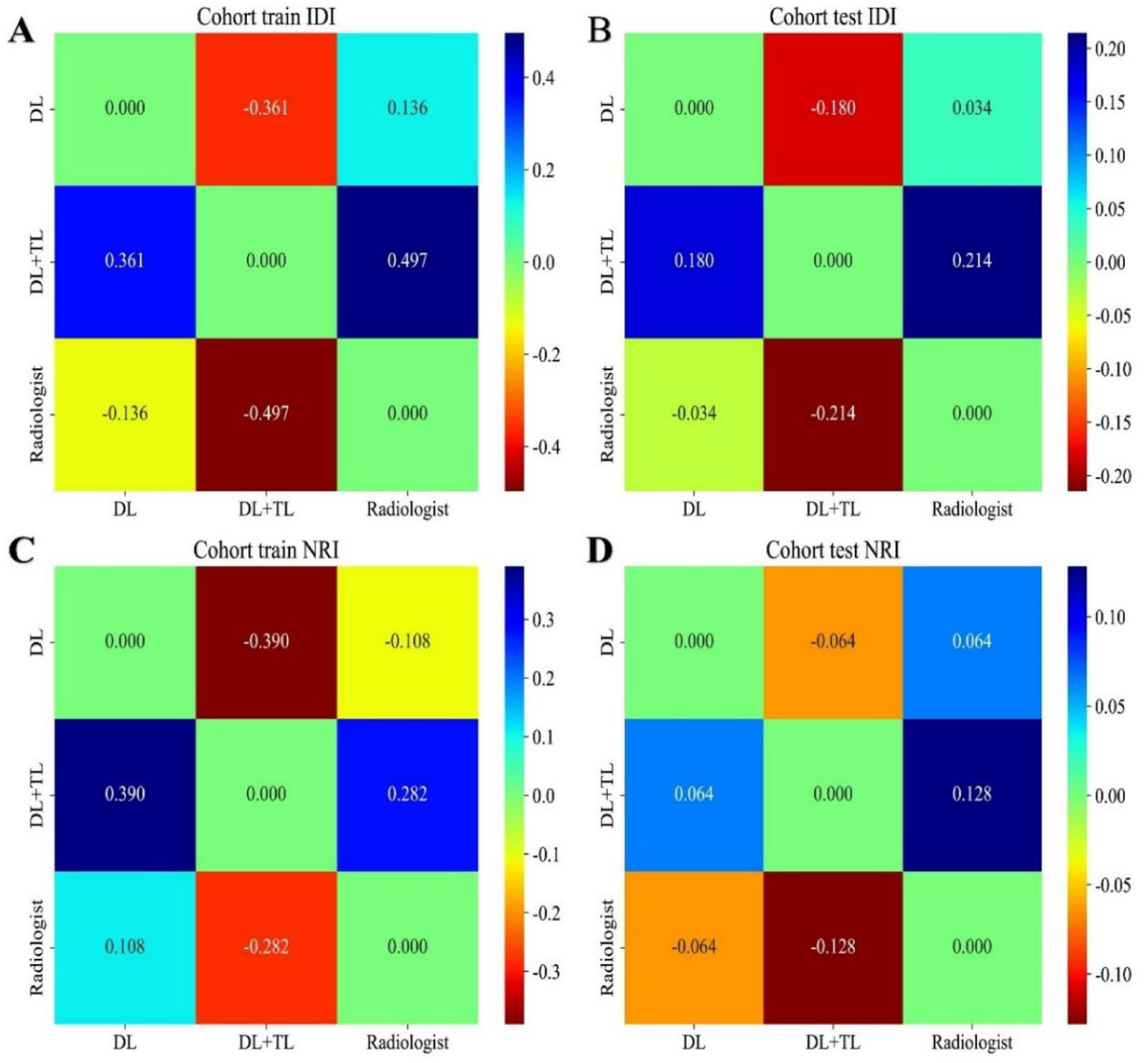
Pairwise comparison of IDI and NRI among DL, DL+TL, and radiologist in training and testing cohorts. **(A,B)** IDI matrices for training **(A)** and testing **(B)**: cells show numeric IDI values; positive indicates better discrimination for the row model versus the column model; color scale at right. **(C,D)** NRI matrices for training **(C)** and testing **(D)**: cells show numeric NRI values; positive indicates net correct reclassification by the row model; color scale at right. All comparisons are pairwise between DL, DL + TL, and Radiologist; color bars differ for IDI and NRI.

NRI (Figures 6A,B) and IDI (Figures 6C,D) analyses in the test cohort showed that the DL + TL model consistently provided the greatest positive values compared to DL and radiologist performance: For IDI: DL + TL vs. DL: 0.18 (DL + TL outperformed DL); DL + TL vs. Radiologist: 0.214 (DL + TL outperformed Radiologist); DL vs. Radiologist: 0.034 (DL slightly outperformed Radiologist). For NRI: DL + TL vs. DL: 0.064 (DL + TL outperformed DL); DL + TL vs. Radiologist: 0.128 (DL + TL outperformed Radiologist); DL vs. Radiologist: 0.064 (DL outperformed Radiologist). The maximum values were observed for the DL + TL model, especially when compared to the radiologist group. Both NRI and IDI analyses showed that the deep learning combined with transfer learning (DL + TL) model significantly outperformed either deep learning alone or the radiologist, demonstrating superior aneurysm identification capability (Table 4).

**Table 4:** Comparison of predictive performance of models.

Model	Cohort	Acc	AUC	95%CI	Sen	Spe	PPV	NPV	Pre	Recall
DL DL + TL	Train	0.845	0.890	0.8091–0.9703	0.680	0.970	0.944	0.800	0.944	0.680
	Train	1.000	1.000	1.0000–1.0000	1.000	1.000	1.000	1.000	1.000	1.000
Radiologist DL	Train	0.845	0.869	0.7782–0.9600	0.960	0.758	0.75	0.962	0.75	0.960
	Test	0.800	0.744	0.5232–0.9640	0.833	0.769	0.769	0.833	0.769	0.833
DL + TL Radiologist	Test	0.840	0.853	0.6990–1.0000	0.667	1.000	1.000	0.765	1.000	0.667
	Test	0.720	0.731	0.5265–0.9350	0.917	0.538	0.647	0.875	0.647	0.917

Metrics include accuracy (Acc), area under the ROC curve (AUC) with 95% confidence interval (95% CI), sensitivity (Sen), specificity (Spe), positive predictive value (PPV), negative predictive value (NPV), precision (Pre), and recall.

As summarized in Table 1, there were no statistically significant differences in demographic and clinical variables between the new training and test sets (*p* > 0.05), confirming that the groups are now comparable.

**Figure 7:**
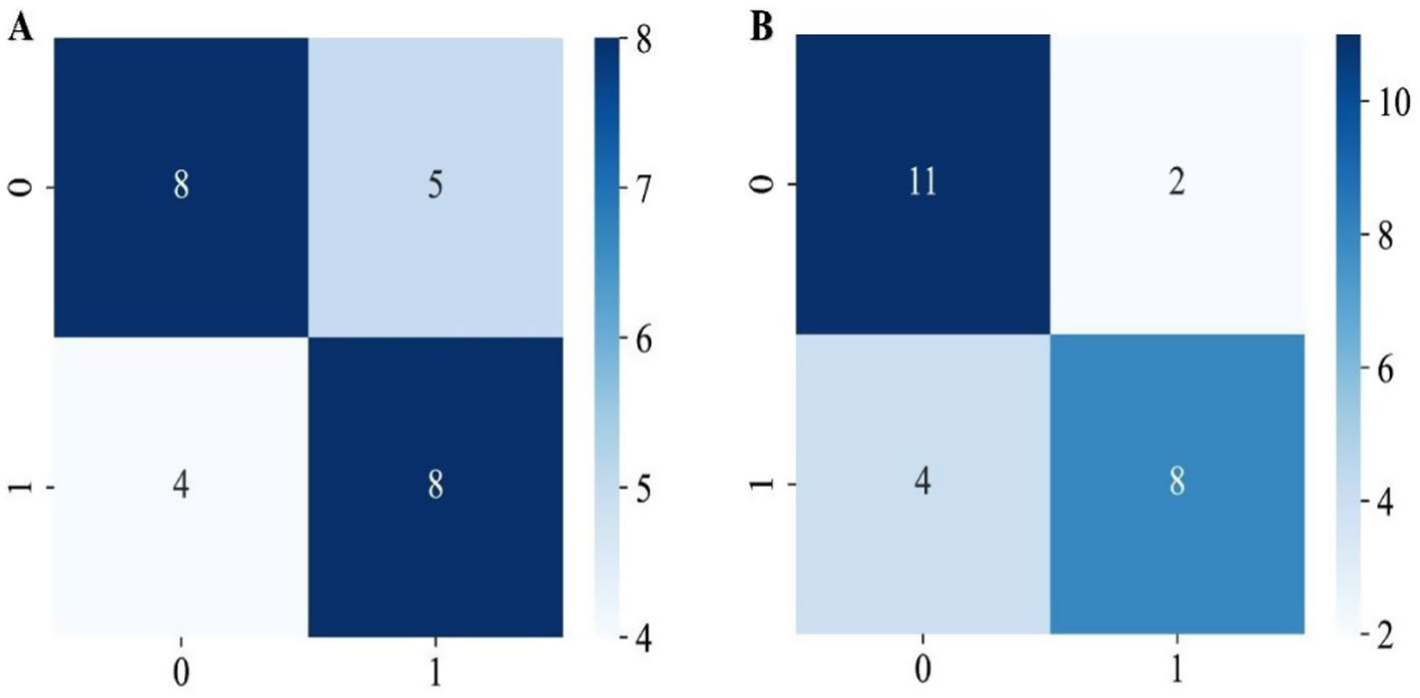
Confusion matrices of model predictions for two cohorts. **(A)** Training cohort: rows = true labels (0, 1), columns = predicted labels (0, 1); numbers in cells are sample counts. **(B)** Testing cohort, same layout. Color intensity reflects count magnitude (color bars at right).

The confusion matrix heatmaps for the two models (Figures 7A,B) visually illustrate the numbers of correctly and incorrectly classified samples. The DL + TL model demonstrated significantly higher accuracy than the DL model (76% vs. 64%), and its specificity was also markedly superior (84.6% vs. 61.5%). The DL + TL model provided more accurate identification of “negative” samples. Both models maintained the same sensitivity for aneurysm detection (66.7%), indicating equivalent ability to identify “positive” cases, but the DL + TL model significantly reduced false positives for “negative” samples.

### 3.3 Interpretability of grad-CAM

**Figure 8:**
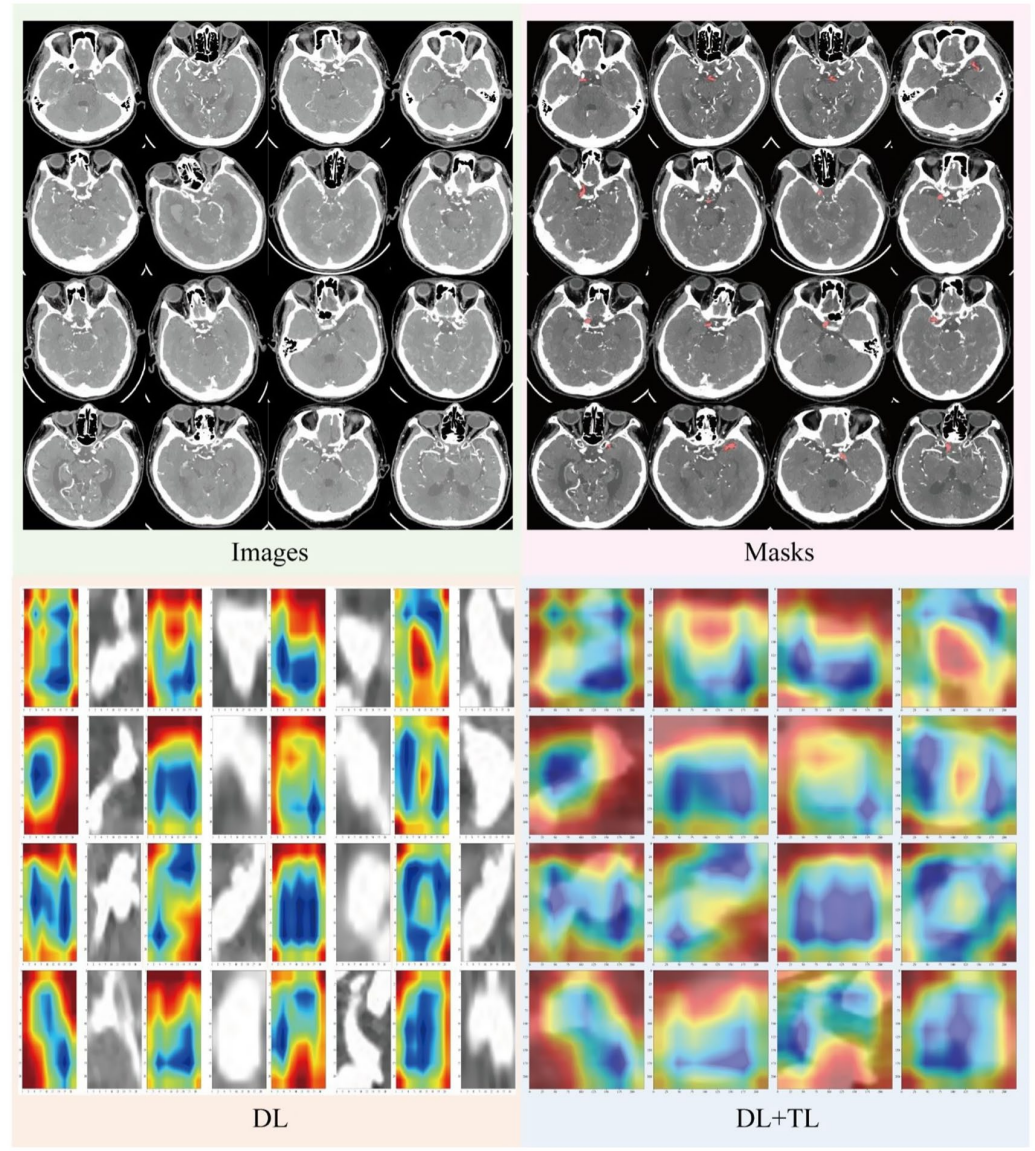
Visualizing model attention via Grad-CAM heatmaps in cranial CT analysis. Representative cranial CT slices, corresponding lesion masks, and Grad-CAM heatmaps illustrate the model’s attention. By adopting a broader yet lesion-specific attention pattern that aligns with expert masks, the model reduces spurious activations in non-ROI regions.

Grad-CAM utilizes gradients to aggregate feature maps without changing the network architecture, and can be applied to various CNN models. To better understand and visualize aneurysm localization, we applied Grad-CAM to both the DL and DL + TL models. Color-coded visualizations (heatmaps, Figure 8) were generated to highlight the focus regions (ROI) attended to by each model when segmenting aneurysms, making the proposed deep learning models more interpretable and aiding clinical decision making. In intracranial aneurysm CT cases, the location of the aneurysm could be clearly identified. The DL model’s attention was relatively diffuse, with a degree of non-specific noise—this “focus scatter” suggests instability in saliency regions. In contrast, the DL + TL model focused its attention more precisely on the aneurysm and its edges, with less noise and fewer irrelevant regions, suggesting that transfer learning enhanced the model’s capacity to identify clinically meaningful features.

The evaluation of Grad-CAM results showed good alignment with expert-annotated aneurysm masks, with a high Intersection-over-Union (IoU) score. A blind evaluation by two independent radiologists indicated that the clinical relevance of the Grad-CAM attention maps was rated highly (mean score = 4.3/5) in most cases. However, there were instances where the Grad-CAM model focused on irrelevant regions, which were identified in the failure case analysis (Table 5). These results suggest that while the model’s interpretability is strong, there are still areas where improvement is needed, particularly in reducing false-positive focus on irrelevant features.

**Table 5:** Grad-CAM evaluation by radiologists and failure case analysis.

Radiologist	Attention map rating (Mean ± SD)	Cases rated (n)	Failure cases identified (n)
Radiologist 1	4.5 ± 0.5	30	2
Radiologist 2	4.1 ± 0.6	30	3
Overall	4.3 ± 0.55	60	5

The Dice similarity coefficient (Dice) is computed using the formula:


$$\text{Dice}=\frac{2\mathrm{TP}}{2\mathrm{TP}+\mathrm{FP}+\mathrm{FN}}$$

Where TP = True Positives, FP = False Positives, and FN = False Negatives.

Alternatively, if Intersection-over-Union (IoU) is available, the Dice coefficient can be derived from IoU using the following relationship:


$$\text{Dice}=\frac{2\times \mathrm{IoU}}{1+\mathrm{IoU}}$$

The IoU between the predicted aneurysm regions and expert annotations was 0.68. Using the formula above:


$$\text{Dice}=\frac{2\times \mathrm{IoU}}{1+\mathrm{IoU}}=\frac{2\times 0.68}{1+0.68}=0.81$$

Thus, the DL + TL model achieved a Dice similarity coefficient of 0.81 ± 0.05, indicating a strong overlap between the predicted and ground truth aneurysm regions. In comparison, the baseline DL model achieved a Dice score of 0.62 ± 0.07, highlighting the improvement in segmentation accuracy when transfer learning (TL) is incorporated.

To enhance the interpretability of our models, we applied Gradient-weighted Class Activation Mapping (Grad-CAM) to visualize the regions of interest (ROIs) that the model focuses on when making its predictions. This technique generates heatmaps that highlight the areas of the input images (in this case, CTA scans) most influential in the model’s decision-making process. These heatmaps provide valuable insights into how well the model is identifying intracranial aneurysms, ensuring that the features contributing to its decisions are both relevant and clinically meaningful.

The Grad-CAM results were evaluated quantitatively using Intersection-over-Union (IoU) and Dice similarity coefficient. For the DL + TL model, the IoU between the predicted and expert-annotated aneurysm regions was 0.68, indicating good overlap. Additionally, the Dice similarity coefficient for the DL + TL model was 0.81 ± 0.05, compared to 0.62 ± 0.07 for the baseline DL model, suggesting that the DL + TL model provided a more accurate delineation of aneurysms. The heatmaps generated by Grad-CAM demonstrated that the DL + TL model’s attention was more focused on the aneurysm regions, whereas the baseline model showed less specificity, with a greater degree of focus scatter.

A blinded evaluation by two independent radiologists rated the clinical relevance of the Grad-CAM heatmaps on a 5-point Likert scale. The DL + TL model’s heatmaps were rated highly, with an average score of 4.3/5, compared to 2.8/5 for the baseline DL model. This supports the superior clinical interpretability of the DL + TL model.

In addition to AUC and accuracy, we evaluated our model’s segmentation performance using the BraTS Dice coefficient, a commonly used metric for assessing the overlap between predicted and ground truth masks in medical image segmentation tasks. Although the BraTS Dice evaluation is primarily used for tumor segmentation in brain MRI, we found it to be relevant in the context of aneurysm detection. Specifically, the Dice coefficient allows us to quantitatively measure how well the model delineates the aneurysm region from the surrounding brain tissue, providing a more granular assessment of model performance.

The Dice coefficient for the DL + TL model was 0.81 ± 0.05, indicating strong overlap with the expert-annotated aneurysm region. This metric complements our AUC and accuracy results by offering a segmentation-specific evaluation, highlighting the model’s ability to accurately localize aneurysms. We believe that this additional evaluation provides useful insights into the model’s segmentation accuracy, which is essential for clinical decision-making in aneurysm diagnosis.

### 3.4 Ablation study

To rigorously assess the contribution of each architectural component within our models, we conducted an ablation study, systematically evaluating the performance of various model configurations. The configurations tested include the baseline ResNet-18 model, ResNet-18 enhanced with LASSO feature selection, ResNet-18 with transfer learning (TL), and the full model incorporating both TL and LASSO with class weighting.

To quantify the performance differences between the various ablation scenarios, we performed paired t-tests across the five folds of the cross-validation. This statistical analysis ensures that the observed differences in performance are statistically significant and not due to random variations. The *p*-values associated with the differences in performance metrics such as area under the curve (AUC) and accuracy are reported in Table 6.

**Table 6:** Ablation study.

Model variant	Transfer learning	LASSO	Class weighting	AUC	Accuracy
ResNet-18 (baseline)	No	No	No	0.71	75%
ResNet-18 + LASSO	No	Yes	No	0.74	78%
ResNet-18 + TL	Yes	No	No	0.81	82%
ResNet-18 + TL + LASSO	Yes	Yes	No	0.84	83%
Full Model (DL + TL + LASSO + weighting)	Yes	Yes	Yes	0.853	84%

To assess the variability of performance across different data splits, we report the standard deviation (SD) and 95% confidence intervals (CI) for each performance metric (AUC and accuracy). These measures provide insight into the consistency of the model’s performance across different folds of the dataset. For instance, the AUC of the DL + TL model was 0.853 ± 0.07, with a 95% CI of [0.789, 0.912], indicating both strong performance and reliable consistency across folds.

All ablation scenarios were evaluated using identical training splits to ensure that the observed performance differences were not influenced by variations in data distribution. The dataset was divided using a 5-fold cross-validation approach, with each fold containing a balanced ratio of aneurysm-positive and aneurysm-negative cases. This approach mitigates potential bias from unequal data distributions across different splits, thereby providing a fair comparison of model performance.

The ablation study provides insight into the specific contribution of each architectural component to the overall performance of the model. The ResNet-18 baseline model exhibited strong performance but was significantly improved when additional components were integrated:

1. LASSO: The inclusion of LASSO regression for feature selection enhanced the model’s ability to eliminate irrelevant features and reduce overfitting, leading to moderate improvements in accuracy and AUC.
2. Transfer Learning (TL): The incorporation of transfer learning, where the ResNet-18 model was pre-trained on ImageNet and fine-tuned on our dataset, led to substantial improvements in both AUC and accuracy. TL enabled the model to generalize better to our small dataset, leveraging learned features from larger datasets and improving performance in detecting aneurysms.
3. Full Model (DL + TL + LASSO + Class Weighting): The most significant improvement was observed in the full model, which combined DL, TL, LASSO feature selection, and class weighting. This integrated approach resulted in a model that not only outperformed the baseline ResNet-18 model but also demonstrated better handling of class imbalance, further improving classification accuracy and discriminative ability. This model achieved an AUC of 0.853 and accuracy of 84%, outperforming the baseline by 0.14 in AUC and 9% in accuracy.

These results demonstrate the complementary contributions of TL and LASSO to improving the performance of the model, with TL enhancing the model’s ability to generalize and LASSO improving feature selection and reducing overfitting.

To evaluate the independent contribution of each architectural component, we conducted an ablation study. Transfer learning provided the largest performance gain (+0.07 AUC), while LASSO-based feature selection provided moderate improvement. The complete pipeline yielded the highest discriminative performance, confirming that each component contributes to overall model robustness.

## 4 Discussion

In this study, we constructed both DL and DL + TL models to comprehensively assess their effectiveness in classifying and recognizing cerebral aneurysms. The results show that the DL + TL model significantly outperformed both standalone DL and radiologist models across multiple evaluation metrics, including ROC curves, decision curve analysis (DCA), integrated discrimination improvement (IDI), and net reclassification improvement (NRI). Grad-CAM visualization further revealed that the DL + TL model’s attention to aneurysm regions was more focused and precise, suggesting that transfer learning enhances the model’s ability to detect key features. This improvement is likely due to the transfer of low-level and high-level semantic features learned from large-scale datasets, mitigating the limitations of small medical imaging datasets and improving generalization and discrimination (25). The original cohort split, which had a significant imbalance between IA-positive and IA-negative cases, was a limitation. The revised results, based on the new balanced split, are more representative of the model’s performance.

An intracranial aneurysm is an arterial dilation resulting from localized vessel wall weakness, and acute mortality can be as high as 40%. Therefore, early diagnosis and timely intervention for intracranial aneurysm is critical, especially periodic monitoring of high-risk individuals to reduce the risk of rupture and its severe consequences (1). Compared with prior aneurysm detection studies, our work further integrates transfer learning with quantitative interpretability assessment and direct radiologist comparison, providing a more comprehensive evaluation framework. (26). Grad-CAM was leveraged for model interpretability analysis, making the “black-box” nature of deep learning models more transparent. This provides a sharper visualization of activation regions for aneurysm localization, highlights subtle morphological changes around the lesion that are of clinical interest, increases diagnostic accuracy, and provides new insights and technical reserves for clinical practice (27). Despite the strong interpretability of the Grad-CAM method, there were several failure cases where the attention maps focused on irrelevant regions of the images. These false positives were identified during the blinded evaluation by the radiologists, who rated the clinical relevance of the Grad-CAM maps. In total, five failure cases were observed. These instances underscore the need for further refinement of the Grad-CAM method to ensure that attention is focused solely on clinically relevant regions. Future work will aim to improve the precision of attention localization and reduce such errors. Despite performance improvements, model complexity remained unchanged, and inference time was clinically feasible (<30 ms per image).

However, this study has several limitations: (1) despite the multicenter data, the limited sample size may not adequately reflect the morphological variability and complex scenarios of aneurysms, which may limit the external generalizability of the model; (2) although model evaluation metrics are comprehensive, further follow-up is required to assess real-world diagnostic utility, such as patient outcomes. Future studies should expand sample size, incorporate multicenter and multimodal data for validation, and further enhance model robustness and generalizability. Continued optimization of transfer learning strategies, evaluation of different CNN architectures, and parameter tuning are also recommended to maximize the benefits of knowledge transfer in studies with small datasets. One of the major limitations of our study is the small sample size (*n* = 83), which may impact the generalizability of the findings. The retrospective design introduces potential biases as the dataset was not randomly assigned, and certain clinical outcomes might be incomplete or unavailable. The small sample size and retrospective nature of the cohort limit the robustness of the conclusions drawn from our results. To address this limitation, future studies should involve larger, prospective cohorts, ideally from multiple centers, to enhance the generalizability and external validity of our model. Furthermore, integrating general neurovascular anatomical knowledge and subtle morphological variations (28–31) via transfer learning, and combining models with Grad-CAM for automated diagnosis, can enhance explainability, increase clinical approval, and improve real-world diagnostic effectiveness (32, 33).

While the model has demonstrated promising performance on the internal dataset from two institutions, external validation is crucial for assessing its generalizability. Unfortunately, due to data-sharing restrictions and logistical challenges, cross-institutional testing could not be performed in this study. The model was trained and validated on a dataset from a limited number of centers, and we acknowledge that this may limit its generalizability to broader clinical settings. Future work should aim to include external validation using multi-institutional datasets, which would help confirm the model’s robustness across different imaging protocols, scanners, and patient populations. This would ensure that the model is not overfitting to the specific characteristics of the current dataset and can generalize well to diverse clinical environments.

We recognize that the model has been validated only on an internal dataset, which introduces potential bias due to the limited number of institutions involved. This may affect the model’s generalizability, particularly when applied to other healthcare settings with different patient demographics, imaging protocols, or clinical practices. The dataset used in this study may also have certain biases, such as the overrepresentation of specific conditions or demographics, which could influence model performance. Therefore, the model’s ability to generalize across diverse real-world clinical scenarios is uncertain, and further validation in broader, multi-center studies is necessary to confirm its robustness in varied healthcare settings.

The dataset used in this study consists of patients from two institutions and includes individuals with a range of clinical conditions, such as a history of ischemic stroke or other neurological deficits. However, this dataset may not fully capture the wide variety of clinical conditions present in the general population, and certain rare or atypical cases may be underrepresented. Additionally, the dataset primarily consists of patients from a specific geographical region, which could limit the model’s performance when applied to populations with different risk profiles or clinical practices (34, 35). To improve the clinical representativeness of the model, future work should focus on expanding the dataset to include a more diverse set of patient conditions, including individuals from different regions, with a broader range of comorbidities and demographic factors.

To address the limitations of the current study, future research should focus on external validation using multi-center data to enhance the model’s generalizability. This will allow for a more robust evaluation of its performance across diverse patient populations, imaging modalities, and clinical conditions. Additionally, efforts to further diversify the clinical dataset will be crucial in improving the model’s applicability in real-world clinical settings. A large-scale prospective study, ideally incorporating multimodal imaging data and including a wider variety of patient demographics, will be critical to further optimizing the model for clinical deployment.

## 5 Conclusion

In this study, we demonstrated that the integration of deep transfer learning (DL + TL) with Grad-CAM visualization techniques demonstrates improved performance the accuracy and interpretability of intracranial aneurysm classification using CT angiography images. Our DL + TL model outperformed both conventional deep learning models and experienced radiologists in multiple evaluation metrics, including AUC, accuracy, calibration plots, decision curve analysis (DCA), net reclassification improvement (NRI), and integrated discrimination improvement (IDI), as well as confusion matrix heatmaps. Furthermore, Grad-CAM provided enhanced visualization of critical aneurysm features, reducing irrelevant focus and supporting more precise clinical decision-making. While limited by its single-center design and relatively small sample size, our research suggests that transfer learning and interpretability tools like Grad-CAM may enhance clinical applicability for advancing neurovascular diagnostics. Future multi-center studies with larger, diverse datasets and further optimization of transfer learning strategies are warranted to maximize clinical utility and generalizability.

## Data Availability

The original contributions presented in the study are included in the article/supplementary material, further inquiries can be directed to the corresponding authors.
